# The unexpected importance of mosquito oviposition behaviour for malaria: non-productive larval habitats can be sources for malaria transmission

**DOI:** 10.1186/1475-2875-4-23

**Published:** 2005-05-13

**Authors:** Arnaud Le Menach, F Ellis McKenzie, Antoine Flahault, David L Smith

**Affiliations:** 1 Fogarty International Center, National Institutes of Health, Bethesda, MD 20892, USA; 2 Université Pierre et Marie Curie, Inserm U707, 27 rue Chaligny, 75012 Paris, France

## Abstract

**Background:**

Mosquitoes commute between blood-meal hosts and water. Thus, heterogeneity in human biting reflects underlying spatial heterogeneity in the distribution and suitability of larval habitat as well as inherent differences in the attractiveness, suitability and distribution of blood-meal hosts. One of the possible strategies of malaria control is to identify local vector species and then attack water bodies that contain their larvae.

**Methods:**

Biting and host seeking, not oviposition, have been the focus of most previous studies of mosquitoes and malaria transmission. This study presents a mathematical model that incorporates mosquito oviposition behaviour.

**Results:**

The model demonstrates that oviposition is one potential factor explaining heterogeneous biting and vector distribution in a landscape with a heterogeneous distribution of larval habitat. Adult female mosquitoes tend to aggregate around places where they oviposit, thereby increasing the risk of malaria, regardless of the suitability of the habitat for larval development. Thus, a water body may be unsuitable for adult mosquito emergence, but simultaneously, be a source for human malaria.

**Conclusion:**

Larval density may be a misleading indicator of a habitat's importance for malaria control. Even if mosquitoes could be lured to oviposit in sprayed larval habitats, this would not necessarily mitigate – and might aggravate – the risk of malaria transmission. Forcing mosquitoes to fly away from humans in search of larval habitat may be a more efficient way to reduce the risk of malaria than killing larvae. Thus, draining, fouling, or filling standing water where mosquitoes oviposit can be more effective than applying larvicide.

## Background

Malaria is responsible for 700,000 to 2.3 million deaths each year, mainly among children [1]. It is caused by four species of *Plasmodium*, protozoan parasites that are most common in the tropics, especially Africa, and are transmitted between humans by the bites of female *Anopheles *mosquitoes. Thus, the distribution of *Anopheles *mosquitoes is an important factor in determining the prevalence of *Plasmodium *infections in humans. At large spatial scales (i.e. 100–1,000 kilometers), the distribution of malaria is best described by climate: warm, humid places with standing water support large mosquito populations and high malaria prevalence. At local scales (i.e. 100 metres to one kilometre), the risk of malaria is determined by mosquito behaviour and ecology, especially the distribution of blood-meal hosts and water. Mosquitoes alternate between blood feeding and oviposition, and suitable hosts and water are heterogeneously distributed [2]. Thus, human biting reflects the mosquitoes' commute to complete its gonotrophic cycle, as well as inherent differences in the attractiveness, suitability and distribution of blood-meal hosts [3]. Here, mathematical models are used as conceptual tools to explore mosquito oviposition behaviour and the availability of water as an explanation for variability in the risk of malaria.

Mathematical models have played an important role in malaria epidemiology. The mathematical models of Ross illustrated the role of mosquitoes in the dynamics of malaria, placing mosquito control at the centre of anti-malaria intervention strategies [4]. The basic concepts of the entomological inoculation rate (EIR), vectorial capacity and the basic reproductive number for malaria (R_0_) were all based on mathematical models of malaria transmission, linking entomology and malaria epidemiology [5]. Several studies and mathematical models have emphasized the role of heterogeneous biting in the dynamics and control of malaria [6,7]. Approximately 20% of the human population contributes 80% of the net transmission of malaria, because mosquitoes bite some people more than others [8].

Heterogeneous biting is due, in part, to the ecology and behaviour of *Anopheles *mosquitoes. A number of surveys, field and lab experiments have been carried out to better understand which cues are responsible for mosquitoes' differential attraction [9]. Mosquitoes emerge from water sources and then fly to a blood-meal host, locating a host using a set of cues, including host movement, odour, CO_2 _and body temperature. Thus, the proximity of households to larval habitat [10], domestic animals [11,12], human avoidance and defensive behaviour [13,14] and individual attractiveness, depending mainly on odour [15,16] or infection status [7], help to explain why some humans are bitten more often than others. Studies have most consistently reported gradients in vector density away from the breeding sites: in the vicinity of the aquatic habitat, the number of adult mosquitoes is higher [17,18] as is malaria prevalence [19,20] without being always correlated with clinical illness [21]. Thus, vector dispersal is driven by the search for oviposition sites as well as the search for hosts: the distance vectors have to fly to lay their eggs influences the radius of control measures [22].

One factor that has been neglected in these studies is the oviposition behaviour of mosquitoes. The cues used by any *Anopheles *species to select the sites at which they oviposit between blood-meals remain poorly understood, except in very general terms. For example, *Anopheles arabiensis *and *Anopheles gambiae *s.s. typically breed in very transient habitats like shallow sunlit fresh water pools or human-made habitats [23], though they may also be common in rice fields [24,25]. In contrast, *Anopheles funestus *breeds mainly in marshes and other types of sheltered habitats that contain vegetation [26,27]. What is suitable one week may become unsuitable the next, due to abiotic (e.g. drying or flooding) or biotic factors (e.g. increased predation or competition). Furthermore, eggs, larval instars and pupae may have different ecological requirements. The standard way to locate the "breeding sites" of malaria vectors is to look for larvae, sample them and identify their species. Often, most of the sites that seem most suitable may be unoccupied by immature *Anopheles *of any stage, at least temporarily. Breeding sites are prone to change, e.g. in accord with agricultural development, deforestation or irrigation [28]. Environmental management [29] allows for vector control focusing on long-term change in vector habitat (draining breeding sites) or on using means that reduce vector reproduction, survival or abundance (i.e. spraying breeding sites with larvicide) [30]. Water source reductions may have played a role in eliminating malaria from Israel, the United States and Italy [31].

It seems clear that larval habitat should be a focal point for malaria transmission, but what are the effects of non-productive water sources where mosquitoes oviposit but eggs fail to develop to adults (e.g. if sprayed with larvicide or desiccated), compared to truly productive water sources (breeding sites)? Does the presence of these unsuitable water sources increase or decrease malaria prevalence? Here, the heterogeneous distribution of water and mosquito oviposition behaviour are explored as factors in heterogeneous biting.

## Methods

### 1. Model

**Figure 1:**
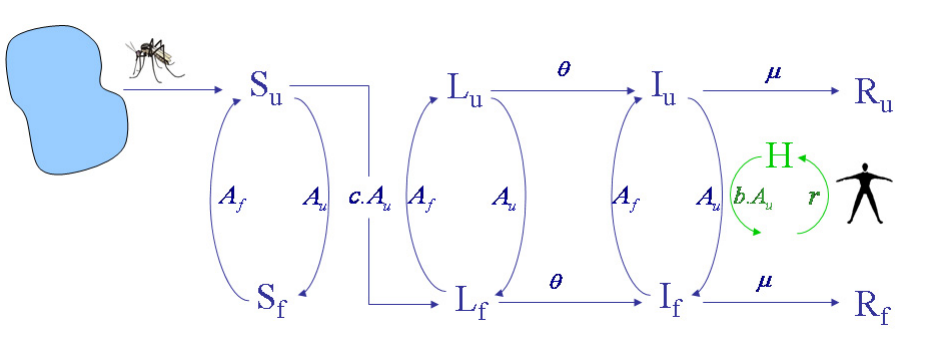
**Malaria transmission dynamics within a patch between mosquito and human population. **Mosquito population (in blue): Mosquitoes emerge from a water source uninfected and unfed (S_u_). Susceptible, unfed mosquitoes feed at rate *A*_*u *_and they are then considered fed and susceptible (S_f_), unless the blood-meal infects the mosquito with malaria (with probability *cX*) in which case they become latent and fed (L_f_). Latent mosquitoes become infectious at the rate θ, regardless of whether they are fed or unfed (*L*_*f *_to *I*_*f*_, or *L*_*u *_to *I*_*u*_). Fed mosquitoes, regardless of their infection status, return to being unfed after ovipositing, at rate *A*_*f*_. (*S*_*u *_to *S*_*f*_, *L*_*u *_to *L*_*f*_, or *I*_*u *_to *I*_*f*_). All mosquitoes die at a rateμ. Human population (in green): Susceptible Human (H) may become infective (X) after an infective bite at a rate *b*.*A*_*u*_. They would return to the susceptible state at a rate *r*.

Here, a recent spatial model for malaria epidemiology on heterogeneous landscapes was modified by incorporating a more detailed description of the gonotrophic cycle into models for mosquito infection dynamics [32] (Figure 1). Let *S *denote the density of susceptible (i.e. uninfected) mosquitoes, *L *denote the density of latent (i.e. in the incubation period: from the onset of infection to the beginning of the infective period) and *I *the density of infective mosquitoes. Whatever her state of infection, the mosquito alternates between the activities of blood-meal feeding and ovipositing. Fed, gravid mosquitoes, denoted with subscript *f*, have taken a blood-meal and seek a place to oviposit, while unfed mosquitoes, denoted with subscript *u*, have recently oviposited and seek a blood-meal host.

Spatial heterogeneity was incorporated by subdividing the landscape into *i *patches in an array or grid (see below), with subscripts denoting the mosquito location and state. Thus, *I*_*i, f *_describes the density of fed, infective mosquitoes in patch *i *(Eq. 1). Let *A*_*i*,*u*_, denote the rate (speed of an event over time) at which unfed mosquitoes feed on a human host in patch *i*, if one is available; upon biting, the mosquito changes state from "unfed" (*u*) to "fed" (*f*). Similarly, let *A*_*i*,*f *_denote the rate at which fed mosquitoes oviposit in patch *i*, if water is available; upon ovipositing, mosquitoes change state from fed to unfed. Thus, if hosts and water are available in patch *i*, the expected time to find a host is *A*_*i*,*u*_^-1^, and the expected time to oviposit is *A*_*i*,*f*_^-1^, so the duration of one gonotrophic cycle in patch *i *is *A*_*i*,*f*_^-1 ^+ *A*_*i*,*u*_^-1^. Note that the human biting rate (HBR) in each patch includes biting only by unfed mosquitoes: *HBR*_*i *_= *A*_*i,u *_(*S*_*i,u *_+ *L*_*i,u *_+ *I*_*i,u*_) / *H*_*i*_, where *H*_*i *_denotes human density in the *i*^*th *^patch. In this model, the entomological inoculation rate (EIR) in each patch includes biting only by infective, unfed mosquitoes, . Similarly, the sporozoite rate in unfed mosquitoes is defined as ratio of unfed infective mosquitoes to total number of unfed mosquitoes .

Let ε_i _denote the local emergence rate of adults. If ε_i _> 0, a patch was considered to be a productive source for mosquitoes. Some patches might have water where mosquitoes can oviposit, but no adults emerge. Thus, if *A*_*i*,*f *_> 0, but ε_i _= 0, the patch was called a non-productive water source. Obviously ε_i _= 0 if no water was available. Thus, it is implicitly assumed that the emergence rate of adult mosquitoes is regulated at the pre-adult stages, not limited by the availability of eggs.

It is assumed that mosquitoes move among patches [33], depending on their gonotrophic state and the availability of hosts and water. Since water or hosts might not be available in some patches, *A*_*i*,*f *_and *A*_*i*,*u *_denote the local biting rates, subject to host availability [34] and local oviposition rates subject to the availability of oviposition habitat [35]. If humans were not available, *A*_*i*,*u*_* = 0*, and if water was not available, *A*_*i*,*f*_* = 0*: a mosquito in an unfed or fed state, respectively, would migrate to another patch in search of a blood-meal host or larval habitat (see below). Thus, the residence time in each disease state is longer if no host or no water is available. Otherwise, the parameters assume a positive value (Table 1). It is assumed that emigration of mosquitoes depends on the presence of water for fed mosquitoes and on human density for unfed mosquitoes. Let *w *denote the per-capita emigration rate of a fed mosquito, which depends on water availability: it describes the expected number of patches a mosquito would cross in one day if no water were available *w *= 0 if water is available). Similarly, it is assumed that the migration of unfed mosquitoes is a function of local human density, *H*_*i*_. Let γ denote the per capita emigration rate of an unfed mosquito; thus γ = ς*e*^-ψ*Hi*^, where ς corresponds to the maximum daily number of patches a mosquito would visit in a day if no humans were available and ψ describes her responsiveness to human density. Another parameterω_*i, j *_describes the proportion of mosquitoes leaving patch *j *that fly into patch *i*; thus, . It is assumed that ω_*i*,*j *_= 0 unless two patches are adjacent and that mosquitoes move in a random direction. The same migration rates were applied for mosquitoes without regard for infection status. Let Ω(*C*_*ij*_) denote the total migration rate for mosquitoes in state *C *and location *i*; for example,  represents the net migration of fed susceptible mosquitoes moving from patch *i*, and  the net migration of unfed susceptible mosquitoes from patch *i*.

**Table 1:** Values, definition and bounds of the parameters used in the model and for the sensitivity analysis (* indicates the parameters used in the multivariate sensitivity analysis). The parameters' values were chosen to mimic an infection by *Plasmodium falciparum *carried by *An. gambiae *s.s. in an adult.

**Symbol**	**Definition**	**Values (bounds)**	**References**
*r*^-1^	Human recovery period	100 days	[47, 48]
*A*_*u*_	Human biting rate	0.5 bites.mosquitoes^-1^.day^-1^	[1, 7, 47]
*b*	Probability that a bite leads to infection among humans	0.5	[49]
*c*	Probability that a bite leads to infection among mosquitoes	0.15	[49]
ς ψ	Migration rate during host seeking	10 patches, 2	(This paper)
w*	Migration rate during oviposition	10 **(1–17) **patches	(This paper)
*A*_*f*_^-1^*	Resting period before oviposition	2 **(1–3) **days	[1, 45, 46]
μ^-1^*	Mosquitoes lifespan	10 **(5–20) **days	[47, 48]
θ^-1^	Incubation period	10 days	[7, 47-49]

Finally, it is assumed that a susceptible mosquito biting an infective human becomes infected with probability *c*, that latent mosquitoes become infective at the rate θ and that mosquitoes die at rate μ. Let *X*_*i *_denote the proportion of humans in patch *i *who are infective, assuming no latency (i.e. no pre-patent period) and no delay between the appearance of merozoites and gametocytes. Let b denote the probability that a bite by an infective mosquito produces a human infection, and r denote the rate at which a human infection is cleared; in other words, it is assumed that the recovery period is exponentially distributed with average duration *r*^-1^. The infection dynamics of malaria in mosquitoes and humans over time and space, including the mosquito gonotrophic cycle, are described by the following equations:



Parameter values are listed in Table 1. The emergence rate was manipulated such that the overall ratio of mosquitoes to humans was 2. The equations were numerically solved over a period of four years; by that date, the system of equations was at the equilibrium . The resulting static spatial distributions of the variables were plotted on two kinds of hypothetical landscapes.

First, simulations of malaria dynamics were performed on a linear array of 17 patches. Humans were uniformly distributed in patches 2 to 16 and absent from the edges. For mosquitoes, three scenarios were considered:

(a) Patch 1 was a productive water source. No water was available elsewhere.

(b) Patch 1 and Patch 9 were productive water sources. No water was available elsewhere.

(c) Patch 1 was a productive water source. Patch 9 was a non-productive water source. No water was available elsewhere.

This landscape is similar to that in a recent study in Tanzania that evaluated mosquito dispersion within three hamlets and showed that marked vectors dispersed differently in relation to the distribution of breeding sites [36].

Second, simulations of malaria dynamics were performed on a square grid of 100 patches (10 × 10). All 10 patches on the left side of the grid were productive water sources (e.g. a stream or pond edge). A non-productive water source was located near the centre of the grid and the human population was uniformly distributed on all the patches except on the left side of the grid. Among the total number of mosquitoes leaving a patch, it was assumed that 80% were randomly flying to patches that shared a side (up, down, left and right patches) and 20% to patches that shared a corner (on the diagonal). This scenario is based on a real-world example: we simulate a village away from a river where the breeding sites are mainly located [37] and with a water source in its centre that is non-productive because it was sprayed with larvicide or prone to desiccation.

### 2. Sensitivity analysis

A sensitivity analysis on the linear array from the scenario (c) above was performed. The evaluation of the influence of water on mosquito migration and distribution focused on the segment of the mosquito population that was fed and seeking a site to oviposit. A multivariate sensitivity analysis [38] to assess the impact of the parameters that describe the behaviour of fed mosquitoes on the proportion of infected humans was performed; parameters examined were the migration parameter *w *(the expected number of patches a mosquito would fly without water), the oviposition parameter *A*_*f*_^-1 ^(the time between taking a blood-meal and oviposition) and mortality μ^-1 ^(mosquito lifespan). These three parameters were sampled in accord with a Latin Hypercube Sampling (LHS) scheme [39]. The distribution of malarial infections in humans was computed using 10,000 sets of parameters drawn from a uniform distribution, with bounds described in Table 1. To evaluate the impact of uncertainty in these parameters we calculated the 5^th ^and 95^th ^percentile of the resulting distribution of the human malaria prevalence for each patch. The Partial Rank Correlation Coefficients (PRCCs) were also calculated using the 10,000 values for each parameter and the 10,000 predicted values in malaria prevalence over each patch. A univariate sensitivity analysis on the three parameters described above was performed using the extreme values.

## Results

**Figure 2:**
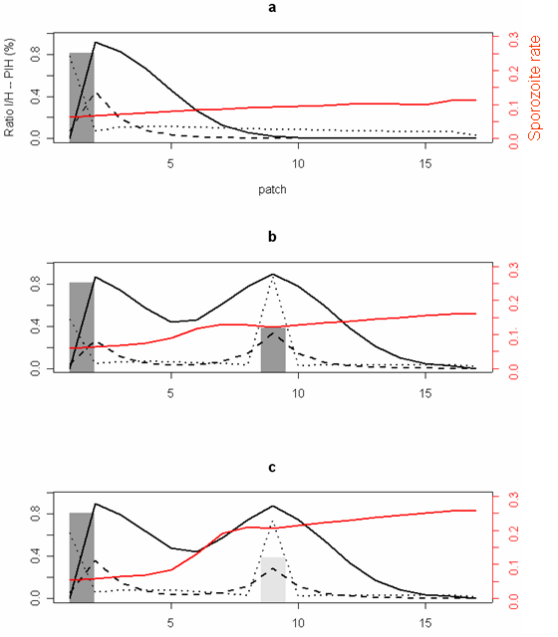
**Distribution of the ratio of infectious mosquitoes (I) to Human (H), of malaria prevalence (PIH: Proportion of infected humans) and of the sporozoite rate for unfed mosquitoes among various aquatic habitats. a) **One productive water source in patch 1. **b) **Two productive water sources in patch 1 and 9. **c) **One productive water source in patch 1 and one non-productive water source in patch 9. Human population was uniformly distributed from patch 2 to 16. The dotted black lines correspond to the ratio of fed infectious mosquitoes (*I*_*f*_) and the dashed black lines to the ratio of unfed infectious mosquitoes *I*_*u*_. The solid black lines correspond to the malaria prevalence (PIH) and solid red lines to the sporozoite rate for unfed mosquitoes. Dark gray bars represent the presence of a productive water source and light gray bars the presence of a non-productive water source.

The distribution of risk along the linear array of patches, as measured by EIR, is related to the distribution of productive water sources and non-productive water sources. EIR is proportional to the density of unfed, infective mosquitoes (see Methods) and thus followed the same distribution (Figure 2). When mosquitoes emerged from a single productive point source (scenario a), EIR peaked in the vicinity of the source (daily EIR = 0.22 in patch 2, Figure 2a). The presence of a second productive water source at an intermediate distance (scenario b) produced a bi-modal distribution of infective mosquitoes (EIR = 0.13 in patch 2 and EIR = 0. 17 in patch 9, Figure 2b). A similar bi-modal distribution was observed when a non-productive water source was located at the same intermediate distance (scenario c, EIR = 0.18 in patch 2 and EIR = 0.14 in patch 9, Figure 2c). Thus, a non-productive water source near a mosquito productive water source acts as a focal point for malaria transmission, even if no adults emerge.

The proportion of infected humans (PIH) followed the same trend as the distribution of EIR. The presence of a productive water source (Figure 2b) or a non-productive water source in patch 9 (Figure 2c) led to similar distributions of the proportion of infected humans, ranging from 85% to 90% in patches 2 and 9. Thus, productive and non-productive water bodies generate similar levels of increased risk of malarial infection. The sporozoite rate in unfed mosquitoes increases slightly with distance from a productive water source (Figure 2a). The presence of a non-productive water source at an intermediate distance results in a substantially increased sporozoite rate (Figure 2c). The difference between the two is the absence of young, non-infectious mosquitoes at the non-productive source.

Among the various scenarios, the overall maximum ratio of infective mosquitoes (fed plus unfed mosquitoes) to humans ranged from 0.85 (patch 2, Figure 2a) to 1.2 (patch 9, Figure 2b) at the maximum point for malaria transmission. In this model, the distributions of fed and unfed mosquitoes differed; overall, the distribution of fed and unfed mosquitoes both reflected the underlying distributions of productive water sources and non-productive water sources. Yet, the ratio of mosquitoes to humans was negatively correlated when water but no humans were available (patches 1 & 2; Figure 2a, b, c); the ratio of fed to unfed mosquitoes is high at the productive water source (Figure 2a; *I*_*f *_= 0.78 in patch 1), and the unfed mosquitoes were mainly in the adjacent patch (*I*_*u *_= 0.44 in patch 2). Thus, no infective unfed mosquitoes were present where only water but no humans were found.

**Figure 3:**
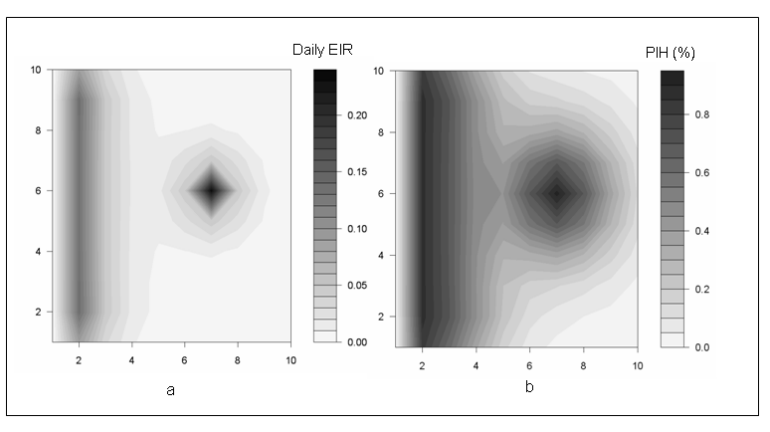
**Malaria risk map in a 10 by 10 grid assumed to be a village: **Productive water sources are located all along the left side of the grid and a non-productive water source is located in the centre of the grid. **a) **The map is based on annual EIR values. **b) **The map is based on the Proportion of Infected Humans (PIH).

Similar patterns emerged on the grid. Two areas were identified as high-risk zones. High values of daily EIR were observed in the patches next to the stream (ranging from EIR = 0.09 to EIR = 0.14). A second high-risk area was focused around the non-productive water source (EIR = 0.23, Figure 3a). Notably, the highest values of EIR were observed at the non-productive water source, not next to the stream where mosquitoes emerged. EIR decreased with the distance from water, whether it was a productive water source or non-productive water source (Figure 3a). Malaria prevalence also reflected EIR. Along the stream, malaria prevalence ranged from 81.8% to 87.2% and at the non-productive water source malaria prevalence was 92.1% (Figure 3b). This underlines a high level of spatial clustering in malaria risk distribution. Note in Figure 3 that the highest variation in malaria prevalence occurred as annual EIR ranged from 0 to 33 and prevalence varied from 0% to 82%; such ranges in EIR values were found over short spatial scales in the simulations, just two patches away from the productive water sources and one patch away from the non productive water source. For larger EIR values, (33 to 86), prevalence changed only 10%, ranging from 82% to 92%.

**Figure 4:**
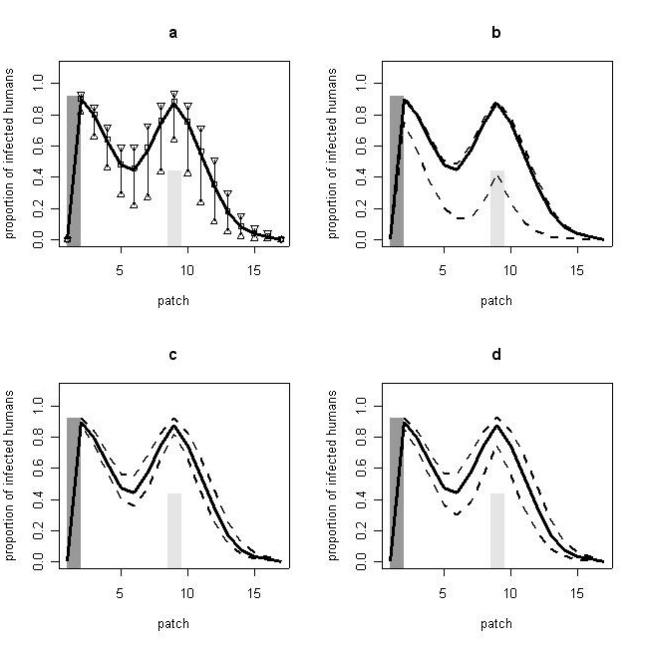
**Proportion of infected humans according to various sets of oviposition parameters**. (The number of empty patches a fed mosquito would fly over each day in order to oviposit, mortality rate of fed mosquitoes, time to oviposit): One productive water source is located in patch 1 and one non-productive water source in patch 9 (scenario c). **a) **Multivariate sensitivity analysis. The thick solid line represents the distribution of the PIH using the realistic values. The triangles represent for each patch the 95^th ^and 5^th ^percentile of the 10,000 simulations using the sets of simulated parameters. The squares represent the median of these 10,000 simulations. **b) **Univariate sensitivity analysis on the maximum number of patches *w*. The dashed lines represent the prevalence distribution for the extreme values (*w *= 1 and *w *= 17) **c) **Univariate sensitivity analysis on the time to oviposit *A*_*f*_^-1^. The dashed lines represent the prevalence distribution for two extreme values (*A*_*f*_^-1 ^= 1 day and *A*_*f*_^-1 ^= 3 days). **d) **Univariate sensitivity analysis on the mortality rate μ^-1^: the dashed lines represent the prevalence distribution for two extreme values (μ^-1 ^= 5 days and μ^-1 ^= 20 days).

The results of the multivariate sensitivity analysis on the linear array demonstrated that the risk of malaria varied, depending on the value of the parameters. The 5^th ^and 95^th ^percentile curves followed the same trends as the point estimate. In the vicinity of the non-productive water source (up to three patches away), malaria prevalence varied up to 49% (figure 4a). Because EIR is sensitive to the oviposition rate, increasing the resting time has a protective effect with respect to malarial infection (PRCCs = -0.18). Increasing the maximum number of patches flown through in search of an oviposition site (PRCCs = 0.27) and increasing lifespan (PRCCs = 0.28) increase the prevalence of malarial infection. All these correlation coefficients were significant (p < 0.0001, t-test). The univariate sensitivity analysis showed the great impact of the maximum mosquito flight distance; at small values, the second peak in the non-productive water source was substantially lower (PIH = 42.5% for *w *= 1 vs. PIH = 87.5% for *w *= 10) (Figure 4b). When mosquitoes flew a longer distance, they would bite along the way and a larger proportion would find and stay at a further water site. The impact of the mosquito lifespan and the oviposition rate had less impact on human prevalence (Figure 4c and 4d).

## Discussion

It has been demonstrated that the availability of water and associated mosquito oviposition behaviour can play an important role in determining the distribution of malaria risk. In some cases, proximity to water where mosquitoes oviposit increases the risk of malaria, whether or not the eggs develop into adults. In other words, a non-productive site for adult mosquito emergence can be a source for malaria. More generally, as would be expected, malaria prevalence would be higher close to water bodies. The transmission potential of mosquitoes is maximized when water and humans are both available.

Because mosquitoes return to water to oviposit, water bodies become a starting point in the search for a blood-meal host. Since mosquitoes fly until they find a host, EIR declines sharply away from water in the model and the heterogeneous distribution of larval habitat produces large variations in EIR over relatively short distances, in agreement with the results from field studies [40]. A review of the literature showed that adult mosquitoes may exhibit high dispersal rates between villages [41] and may fly up to 5 km, but half of the flights were within a 1 km radius [42]. An analysis encompassing surveys from all over Africa showed that annual EIR ranged from 0 to 702 and the malaria prevalence from 7% to 94.5% and that, as in this model, beyond a threshold, increases in EIR value did not affect malaria prevalence [43,44].

The parameter values were consistent with studies of *Anopheles *species, but substantial variation exists among species, locations and at the same location over time [1,7,45-49]. The aim of the research was to investigate the influence of oviposition behaviour on the spatial distribution of infective mosquitoes; substantial uncertainty remains about strategic aspects of mosquito behaviour, such as how mosquitoes locate and choose a place to oviposit. Many cues could make mosquitoes oviposit in non-productive water sources, including larval crowding and the ability of a mosquito to detect it, the inability to detect unsuitable habitat, habitat desiccation, wind, mosquito physiological status, and many other factors. The search for water in which to oviposit may be a sensitive point in the gonotrophic cycle because the mosquitoes are heavier, with higher energy expenditures and perhaps higher mortality. Thus, increases in flight distances for gravid mosquitoes may increase per-capita mortality rates and thus diminish transmission capacity. Another interesting result involved the effect of the maximum flight distances during this search for an oviposition site. For short flight distances, mosquito distributions reflected the distribution of larval habitat, true productive water sources. As flight distances increased, mosquito distributions resembled the distribution of water, including non-productive water sources as well as productive water sources (data not shown). However, increasing maximum flight distances beyond a threshold (about eight patches per day) had little impact on the distribution of EIR.

This research focused on the ecology and behaviour of *Anopheles *mosquitoes, and how the heterogeneous distribution of water bodies influences the heterogeneity in their biting. Mosquito populations fluctuate with weather and climate, increasing in the wet season and decreasing in the dry season [50]. Seasonality was ignored to better focus on the impact of oviposition behaviour on the risk of malaria transmission. For the same reason heterogeneous human populations were not considered [51]. Oviposition is one of many factors determining the distribution of risk, but it should be considered as a possible reason for mosquito aggregation, one that would interact with other factors such as seasonality and heterogeneous human distributions. For example, the distribution of oviposition habitat may become more heterogeneous during the dry season, leading to increased mosquito aggregation around water. The availability of larval habitat is sometimes correlated with household density as the number of breeding sites may increase with density up to some threshold [52]. Finally, mosquito memory may limit oviposition in unsuitable habitats [53]. It has been demonstrated that mosquito dispersal might be restricted by a tendency to return to known locations for oviposition, that is productive water sources. Nevertheless, even if vector learning counterbalances the possibility that mosquitoes oviposit in non-productive sites, short vector life spans and the ephemeral nature of suitable larval habitat make aggregation around non-productive sites a potentially important factor. In future research, model refinements could include improvements to the representation of vector biology by adding an explicit resting compartment and different mortality rates as functions of vector disease status. These refinements would further improve our understanding of how far mosquito dispersal can be observed and how large a control area should be explored.

Based in part on Macdonald's mathematical models, the Global Malaria eradication campaign focused on increasing adult *Anopheles *mortality using DDT and more specifically on reducing adult survival rates [47]. Some earlier workers had also emphasized the importance of distinguishing vector from non-vector species and identifying their actual breeding sites, so that targeted, sustainable anti-vector programs could replace ineffective or inefficient generalized anti-mosquito approaches. Ross used a mathematical model to conclude that, "...in order to counteract malaria anywhere we need not banish *Anopheles *there entirely...we need only to reduce their numbers below a certain figure" [54]. Though few of his contemporaries paid attention to this idea of threshold densities of *Anopheles*, some applied it in successful programs of environmental management [55]. Eliminating water in the neighbourhood of humans would force mosquitoes to commute longer distances, decreasing the human feeding rate and increasing mortality during the extrinsic incubation period, hence, decreasing vectorial capacity. Because a non-productive water body can be a source for malaria, an intervention that eliminates water where mosquitoes may oviposit, or fouls the water to deter oviposition, would be more effective for malaria control than using larvicide to reduce mosquito density. Even though larvicides may have adverse effects by killing larval predators [56], treating distant water sources with larvicide might provide a complementary control strategy. This concept is similar to zooprophylaxis [57]; it has been argued that cattle, treated with insecticide or not, provide efficient control if situated between humans and larval habitat and far enough from dwellings [58].

## Conclusion

The approach previously described provides a framework for mapping the risk of malaria based on fine-grained maps of water and humans [59]. Such methods provide a tool for mapping risk and planning intervention. An important issue in developing these maps is to identify which biological details are necessary to include and which details can be omitted. These models suggest that malaria risk is highest in the vicinity of water where mosquitoes oviposit, a useful observation with great public health implications if true productive larval habitat is harder to identify.

## Authors' contributions

ALM refined the model, performed the sensitivity analysis, carried out the simulations and wrote the manuscript. FEM actively participated in the follow-up of the study and helped to draft the manuscript. AF helped to draft the manuscript. DLS designed and supervised the study, built the model and helped to write the manuscript.
